# Sexual Activity and Impairment in Women with Systemic Sclerosis Compared to Women from a General Population Sample

**DOI:** 10.1371/journal.pone.0052129

**Published:** 2012-12-14

**Authors:** Brooke Levis, Andrea Burri, Marie Hudson, Murray Baron, Brett D. Thombs, Canadian Scleroderma Research Group (CSRG)

**Affiliations:** 1 Lady Davis Institute for Medical Research, Jewish General Hospital, Montréal, Québec, Canada; 2 Department of Twin Research and Genetic Epidemiology, King's College, London, United Kingdom; 3 Psychopathology and Clinical Intervention, Institute of Psychology, University of Zürich, Zürich, Switzerland; 4 Department of Medicine, McGill University, Montréal, Québec, Canada; 5 Department of Psychiatry, McGill University, Montréal, Québec, Canada; 6 Department of Epidemiology, Biostatistics, and Occupational Health, McGill University, Montréal, Québec, Canada; 7 Department of Educational and Counselling Psychology, McGill University, Montréal, Québec, Canada; 8 School of Nursing, McGill University, Montréal, Québec, Canada; University of Pittsburgh, United States of America

## Abstract

**Objective:**

Reports of low sexual activity rates and high impairment rates among women with chronic diseases have not included comparisons to general population data. The objective of this study was to compare sexual activity and impairment rates of women with systemic sclerosis (SSc) to general population data and to identify domains of sexual function driving impairment in SSc.

**Methods:**

Canadian women with SSc were compared to women from a UK population sample. Sexual activity and, among sexually active women, sexual impairment were evaluated with a 9-item version of the Female Sexual Function Index (FSFI).

**Results:**

Among women with SSc (mean age = 57.0 years), 296 of 730 (41%) were sexually active, 181 (61%) of whom were sexually impaired, resulting in 115 of 730 (16%) who were sexually active without impairment. In the UK population sample (mean age = 55.4 years), 956 of 1,498 women (64%) were sexually active, 420 (44%) of whom were impaired, with 536 of 1,498 (36%) sexually active without impairment. Adjusting for age and marital status, women with SSc were significantly less likely to be sexually active (OR = 0.34, 95%CI = 0.28–0.42) and, among sexually active women, significantly more likely to be sexually impaired (OR = 1.88, 95%CI = 1.42–2.49) than general population women. Controlling for total FSFI scores, women with SSc had significantly worse lubrication and pain scores than general population women.

**Conclusions:**

Sexual functioning is a problem for many women with scleroderma and is associated with pain and poor lubrication. Evidence-based interventions to support sexual activity and function in women with SSc are needed.

## Introduction

Systemic sclerosis (SSc), or scleroderma, is a chronic, multi-system, connective tissue disorder characterized by abnormal fibrotic processes and excessive collagen production, which manifests itself in skin thickening and fibrosis of internal organs [1]. Approximately 80% of SSc patients are women, with highest onset rates between ages 30–60 [2]. Common causes of disability include limitations in physical mobility, pain, fatigue, depressive symptoms, and body image distress from disfigurement [3]–[8].

In the general population, sexual activity and impairment rates are, among other factors, highly associated with age and marital status [9], [10]. For instance, in a large population study of over 3,000 women from the metropolitan Boston area, the adjusted odds of being sexual active were approximately 3 times as high for women in the 30–39 age group than for women aged 50–59. Among sexually active women, on the other hand, the odds of impairment were more than 3 times as high in women 50–59 as among women 30–39. The odds of sexual activity among married women were approximately 6 times the odds for unmarried women, although married women who were active were more likely to be sexually impaired compared to sexually active unmarried women.

In women with SSc, physical and psychological consequences of the disease, including fatigue, depression, disfigurement, Raynaud's phenomenon, skin tightening and discomfort, vaginal tightness and dryness, thickening of skin around the lips, painful finger ulcers and calcium deposits, gastrointestinal symptoms, joint pain and muscular weakness, may affect sexual function [11]–[16]. A recent study found that only 41% of 547 female SSc patients in the Canadian Scleroderma Research Group (CSRG) Registry reported sexual activity in the past 4 weeks [12]. Over 60% of sexually active patients reported impaired sexual function based on the short version of the Female Sexual Function Index (FSFI) [12], [17]. Overall, only 17% of patients were sexually active without impairment. In multivariate analysis, women who were sexually active were significantly more likely to be younger, and to have fewer gastrointestinal symptoms and less severe Raynaud's phenomenon symptoms. Women who were sexually impaired were significantly more likely to be older and to have greater skin involvement and more severe breathing problems. Disease duration was unrelated to sexual activity and impairment.

Limited sexual activity and impaired sexual function appear to be common among women with many chronic illnesses [18], including SSc [11], [12], [19]–[23]. We do not know of any studies, however, that have compared activity and impairment rates among women with a chronic illness to general population data. Thus, the objectives of this study were to 1) compare sexual activity and impairment rates, stratified by age and marital status, between women with SSc and women from a population sample; 2) estimate the overall odds of being sexually active and of experiencing impairment for women with SSc compared to the population sample, controlling for age and marital status; and 3) identify domains of sexual function most strongly related to impairment in women with SSc.

## Methods

There are no Canadian population studies that have used the FSFI to assess sexual activity and impairment. Thus, this study involved a secondary analysis of existing databases of women with SSc from the CSRG Registry and a general population sample from the Adult Twins UK registry [9].

### Ethics Statement

Ethics approval for the present study was obtained from the Research Ethics Board of the Jewish General Hospital, Montreal, Canada. The CSRG Registry was approved by the McGill University Institutional Review Board and the research ethics boards of each participating CSRG site. All CSRG Registry patients provided informed written consent. The sexual functioning study for the Twins UK sample was approved by the St. Thomas' Hospital Research Ethics Committee and all participants provided informed written consent.

### SSc Patient Sample

Female patients from 12CSRG Registry sites from across Canada completed study questionnaires between July 2008 and February 2012. Eligible Registry patients are ≥18 years old, have a rheumatologist-confirmed diagnosis of SSc, and are fluent in English or French. At annual Registry visits, patients undergo extensive clinical evaluations and complete self-report questionnaires, including questions on sexual function.

### UK Population Sample

This sample consisted of monozygotic and dizygotic female twin individuals enrolled in the Adult Twins UK Registry who completed the FSFI in 2008 [24]. Registry participants were recruited through successive national media campaigns in the United Kingdom and Ireland and from other twin registers, including the Aberdeen Twin Registry and the Institute of Psychiatry Adult Registry. The Twins UK sample has been found to be similar to other UK female population samples in terms of disease prevalence and lifestyle characteristics [25]–[27].

### Measures

#### Sexual Activity

In the CSRG Registry, women were classified as sexually active/inactive based on the question, “*During the past 4 weeks, have you engaged in sexual activities with your partner?*” In the UK population sample, rather than a single question, classification was made based on the response “*no sexual activity*” in the past 4 weeks, which was included as a response on 7 FSFI questions (of 9 total FSFI questions).

#### Sexual Impairment

Studies on female sexual function have been criticized for coding sexually inactive women as impaired [28]. Thus, in both samples, sexual impairment was only assessed among sexually active women. A 9-item abbreviated version [12] of the 19-item FSFI, which assesses sexual activity and functioning over the past 4 weeks [17] was used. The 9-item abbreviated version includes items assessing 5 dimensions of sexual function, including desire (2 items), arousal (1 item), lubrication (1 item), orgasm (3 items), and pain (2 items). We included women who responded to items from all domains, and who were missing ≤1 item from any domain and ≤3 items total.

Items on the FSFI are scored from 1–5 with the exception of 2 items related to pain during and following vaginal intercourse, which are scored 0 if vaginal intercourse was not attempted. The original FSFI has good reliability and validity and differentiates between women with and without sexual dysfunction diagnoses [17], [29]–[31]. A 10-item abbreviated version correlated highly with the original 19-item version (*r* = 0.98) in a sample of 568 women [10], [29]. The only difference between the 10-item version and the 9-item version used in this study is that the 9-item version included 2 pain items, rather than 3. In previous studies, the 3 pain items produced substantively identical mean scores and very high estimates of internal consistency (3-item Cronbach's alpha  = 0.94–0.98) [17], [29]–[31], suggesting item redundancy and that weighted total scores of the 9-item and 10-item versions would be comparable since the difference in number of items is adjusted for by domain weighting. To obtain a full-score on the FSFI, domain scores are weighted and summed [12], [17]. A cut-off score of 22.5 was used to classify impairment/non-impairment. This cut-off effectively differentiates women with and without sexual dysfunction based on DSM-IV criteria [10].

#### Sexual Satisfaction

Sexual satisfaction was assessed among sexually active women using the question, “*Over the past 4 weeks, how satisfied have you been with your overall sex life*?” Responses were on a 1–5 scale from “very satisfied” to “very dissatisfied”.

#### Marital Status

In the CSRG Registry, women were classified as married if they indicated being married or living as married. In the UK population sample, women were classified as married if they indicated being married or being in a relationship and living with their partner.

#### Education level

Education level obtained was based on self-report and classified as “≤ High School” or “> High School.” In the CSRG sample, patients identified the highest level of education they had received and responses were dichotomized as “≤ High School” or “> High School.” In the UK population sample, participants identified the number of years of schooling they had received, and a cut-off of 11 years was used to dichotomize responses as “≤ High School” or “> High School”.

#### Clinical characteristics (CSRG sample only)

Time since SSc diagnosis and time from first non-Raynaud's disease manifestation were recorded by study physicians. Skin involvement was assessed using the modified Rodnan skin score [32], a widely used clinical assessment where the examining rheumatologist records the degree of skin thickening from 0 (no involvement) to 3 (severe thickening) in 17 body areas (total score range 0–51). Patients were classified into limited and diffuse cutaneous subsets based on Leroy's definition [33].

**Table 1:** Comparison of sociodemographic and clinical characteristics of women with systemic sclerosis and women from a UK general population sample.

Sociodemographic Characteristics	Systemic Sclerosis Patients (N = 730)	UK General Population Sample (N = 1,498)	P Value
Age in years, *mean (standard deviation)*	57.0 (11.3)	55.4 (11.5)	0.001
Education, *n (%)*:			<0.001
≤ High School	356 (49)	992 (66)	
> High School	373 (51)	344 (23)	
Not reported	1 (0.1)	162 (11)	
Marital Status, *n (%)*:			<0.001
Married or Living as Married	505 (69)	877 (59)	
Not Married	225 (31)	621 (41)	
Clinical Characteristics			
Time since non-Raynaud's symptom onset in years, *mean (standard deviation)*(N = 720)	12.8 (9.7)	----------	
Time since diagnosis of SSc in years, *mean (standard deviation)*(N = 722)	10.0 (8.6)	----------	
Modified Rodnan skin score, *mean (standard deviation)*(N = 706)	8.0 (8.4)	----------	
Diffuse SSc, *n (%)*(N = 681)	171 (25)	----------	

### Data Analyses

The percentages of women with SSc and women from the general UK population who reported being sexually active were calculated, and rate ratio analyses were conducted, stratified by age group and marital status. Among those reporting being sexually active, rates of sexual impairment (FSFI total ≤22.5) were compared similarly across samples by age and marital status.

Multivariate logistic regression analyses were used to assess the independent contributions of sample group (CSRG or UK general population), age in years and marital status to sexual activity status and impairment status. Post-hoc analyses including education level as an additional variable in the regression models were also performed. In addition, separate multivariate logistic models were run to compare the subset of patients with limited SSc versus the general population sample, and then the subset of patients with diffuse SSc versus the general population sample.

Discrimination and calibration of the logistic regression models were assessed with the c-index and Hosmer-Lemeshow goodness-of-fit test statistic (HL), respectively [34]. The c-index is the percentage of comparisons where sexually active (or sexually impaired) patients had a higher predicted probability of being sexually active (or sexually impaired) than inactive patients (or non-impaired patients), for all possible pairs of active and inactive patients (or impaired and non-impaired patients). The HL is a measure of the accuracy of the predicted number of cases of active or impaired patients compared to the number of patients who actually reported sexual activity or impairment across the spectrum of probabilities. A relatively large p value indicates that the model fits reasonably well.

In order to identify areas of sexual function that are particularly problematic for women with SSc, sexual domain scores were calculated among women who were sexually active, and analysis of covariance was used to assess the differences in each sexual domain score between women with SSc and women from the general population sample, controlling for total FSFI scores. Analyses were also performed using Pearson's correlations to determine the correlation between domain scores for the domains that were found to have significantly worse scores among women with scleroderma compared to the general population. This was done to assess the degree to which important problem areas for women with SSc seemed to represent general disease severity versus specific problems that may be independent of each other. Finally, among sexually active women in both samples, Pearson's correlations were used to assess the association between FSFI total and individual sexual domain scores and sexual satisfaction.

All analyses were conducted using SPSS version 20.0 (Chicago, IL), and statistical tests were 2-sided with a *P*<0.05 significance level.

## Results

### Sample Characteristics

There were 800 women with SSc and 1,589 women from the UK general population sample who completed questionnaires. Of these, 44 women with SSc and 84 from the UK did not indicate their sexual activity status. Among sexually active women, 16 with SSc and 7 from the UK did not have complete data for sexual impairment analyses. A further 10 women with SSc did not indicate their marital status. Thus, there were 730 women with SSc (91%) and 1,498 women from the UK (94%) with complete data included in analyses.

Sociodemographic characteristics for both samples and clinical characteristics for the SSc sample can be found in Table 1. The SSc sample had a mean age of 57.0 (SD = 11.3; range 18–83), and the UK sample had a mean age of 55.4 (SD = 11.5; range 25–82; p = 0.001). Almost 70% of women with SSc were married, compared to just under 60% in the UK sample (p<0.001). Women with SSc were also more likely to have at least high school education (p<0.001). Among women with SSc, 75% had limited cutaneous SSc, and mean time since onset of non-Raynaud's symptoms was approximately 10 years.

Women with SSc who were not included in analyses due to missing data (n = 70) were slightly older (mean age 61.7, SD = 13.2), less likely to be married (62% married), and less likely to have at least a high school education (34% > high school education), compared to women with complete data, but had similar clinical characteristics. Among women in the UK sample, women who were not included in analyses (n = 91) were, similarly to in the SSc sample, older (mean age 65.5, SD = 9.3), less likely to be married (46% married), and less likely to have at least a high school education (18% > high school education).

Overall, 296 women with SSc (41%) were sexually active, 181 (61%) of whom were sexually impaired. In the population sample, 956 women (64%) were sexually active, 420 (44%) of whom were sexually impaired. Thus, taken as a whole, 115 of 730 women with SSc (16%) were sexually active without impairment, compared to 536 of 1,498 of women from the UK general population sample (36%).

### Sexual Activity in SSc Compared to Population Sample

Rates of sexual activity, stratified by age group and marital status, are presented in Table 2. There were relatively few women with SSc in the 18–29 and 30–39 age groups for both married and unmarried women. Among all other age group/marital status combinations, women with SSc were significantly less likely to be sexually active than women from the UK population sample.

**Table 2:** Comparison of sexual activity rates between women with systemic sclerosis and women from a UK general population sample, stratified by age and marital status.

	Married						Non-Married					
	CSRG		UK				CSRG		UK			
Age Group	N	N (%) Active	N	N (%) Active	Rate Ratio	95% CI	N	N (%) Active	N	N (%) Active	Rate Ratio	95% CI
**18–29**	5	4 (80)	8	5 (63)	1.28	0.64–2.56	8	2 (25)	10	8 (80)	0.31	0.09–1.08
**30–39**	18	12 (67)	91	81 (89)	0.75	0.54–1.05	13	7 (54)	65	52 (80)	0.67	0.40–1.13
**40–49**	103	71 (69)	143	119 (83)	0.83	0.71–0.96	36	14 (39)	110	88 (80)	0.49	0.32–0.74
**50–59**	171	92 (54)	294	220 (75)	0.72	0.62–0.84	52	9 (17)	186	117 (63)	0.28	0.15–0.50
**60–69**	156	62 (40)	263	140 (53)	0.75	0.60–0.93	79	10 (13)	172	78 (45)	0.28	0.15–0.51
**70+**	52	11 (21)	78	34 (44)	0.49	0.27–0.87	37	2 (5)	78	14 (18)	0.30	0.07–1.26
**Total**	505	252 (50)	877	599 (68)	0.73	0.66–0.81	225	44 (20)	621	357 (57)	0.34	0.26–0.45

In multivariate logistic regression analysis, older age was significantly associated with a lower likelihood of sexual activity (odds ratio [OR] = 0.94, 95% confidence interval [CI] = 0.93–0.94, *P<*0.001), while those who were married were more likely to be sexually active (OR = 2.21, 95% CI = 1.82–2.68, *P<*0.001). Controlling for age and marital status, women with SSc were significantly less likely to be sexually active than women from the general population sample (OR = 0.34, 95% CI = 0.28–0.41, *P<*0.001). The model had adequate discriminative power (c-index = 0.742) and calibration (*P = *0.288 for the HL statistic).

In post-hoc sensitivity analyses, women with more than a high school education level were significantly more likely to be sexually active (OR = 1.26, 95% CI = 1.02–1.57, *P* = 0.035). Inclusion of the education variable in the modeldid not substantively influence the assocation of any of the other variables in the core model with sexual activity. When separate models were run for patients with limited and diffuse SSc, patients with limited SSc were significantly less likely to be sexually active than women from the general population sample (OR = 0.33, 95% CI = 0.26–0.42, *P<*0.001), controlling for age and marital status, as were patients with diffuse SSc (OR = 0.37, 95% CI = 0.27–0.53, *P<*0.001).

### Sexual Impairment in SSc Compared to Population Sample

Rates of sexual impairment (among sexually active women) stratified by age group and marital status, can be found in Table 3. There were small numbers of women with SSc in each age group who were unmarried, as well as small numbers who were married and under age 40. For each age group of married women aged 40 and above, women with SSc were significantly more likely to be sexually impaired than women from the UK population sample.

**Table 3:** Comparison of sexual impairment rates between women with systemic sclerosis and women from a UK general population sample, stratified by age and marital status.

	Married						Non-Married					
	CSRG		UK				CSRG		UK			
Age Group	N	N (%) Impaired	N	N (%) Impaired	Rate Ratio	95% CI	N	N (%) Impaired	N	N (%) Impaired	Rate Ratio	95% CI
**18–29**	4	3 (75)	5	4 (80)	0.94	0.46–1.92	2	0 (0)	8	1 (13)	0	-----
**30–39**	12	7 (58)	81	23 (28)	2.05	1.14–3.71	7	5 (71)	52	14 (27)	2.65	1.39–5.07
**40–49**	71	36 (51)	119	41 (34)	1.47	1.05–2.06	14	5 (36)	88	16 (18)	1.96	0.86–4.51
**50–59**	92	60 (65)	220	112 (51)	1.28	1.05–1.56	9	6 (67)	117	52 (44)	1.50	0.91–2.48
**60–69**	62	45 (73)	140	79 (56)	1.29	1.04–1.59	10	5 (50)	78	42 (54)	0.93	0.48–1.78
**70+**	11	8 (73)	34	26 (76)	0.95	0.63–1.43	2	1 (50)	14	10 (71)	0.70	0.17–2.91
**Total**	252	159 (63)	599	285 (48)	1.33	1.17–1.50	44	22 (50)	357	135 (38)	1.32	0.96–1.83

In multivariate logistic regression analysis among sexually active women, older women were more likely to be sexually impaired, (OR = 1.05, 95% CI = 1.04–1.06, *P<*0.001), as were those who were married (OR = 1.41, 95% CI = 1.09–1.82, *P = *0.009). Controlling for age and marital status, women with SSc were significantly more likely to be sexually impaired than women from the general population sample (OR = 1.88, 95% CI = 1.42–2.49, *P<*0.001). The model had adequate discriminative power (c-index = 0.668) and calibration (*P = *0.190 for the HL statistic).

When education was included in the model, women with more than a high school education level were significantly more likely to be sexually impaired (OR = 1.40, 95% CI = 1.07–1.84, *P* = 0.015). Inclusion of the education variable in the model, however, did not substantively influence the assocation of any of the other variables in the core model with sexual impairment. Patients with limited SSc were significantly less likely to be sexually active than women from the general population sample (OR = 1.97, 95% CI = 1.41–2.75, *P<*0.001), controlling for both age and marital status, as were patients with diffuse SSc (OR = 2.15, 95% CI = 1.32–3.51, *P = *0.002).

### Comparison of Sexual Domains in SSc and Population Sample

Differences in sexual domain scores between women with SSc and women from the population sample, unadjusted and adjusted for total FSFI score, can be found in Table 4. For all domains of sexual functioning, women with SSc had significantly worse scores, indicating greater impairment, than women from the general population. Controlling for total FSFI scores, pain and lubrication scores were significantly worse for women with SSc. Pain and lubrication scores were more highly correlated amongst women with SSc (r = 0.62, *P*<0.001) than among women from the population sample (r = 0.36, *P*<0.001).

**Table 4:** Comparison of FSFI domain scores between sexually active women with systemic sclerosis Patients and sexually active women from a UK general population sample; unadjusted and adjusted for total FSFI score.

	Unadjusted Domain Scores			Domain Scores, Adjusted for Total FSFI Score		
FSFI Domain	Mean Difference (UK – CSRG)	P value	Hedge's g	Mean Difference (UK – CSRG)	P value	Hedge's g
**Desire**	0.29	<0.001	0.25	−0.11	0.054	−0.13
**Arousal**	0.22	0.014	0.16	−0.31	<0.001	−0.38
**Lubrication**	0.94	<0.001	0.66	0.40	<0.001	0.43
**Orgasm**	0.36	0.001	0.25	−0.19	0.003	−0.20
**Pain**	0.75	<0.001	0.46	0.21	0.012	0.17

(CSRG Sample: N = 296; UK Sample: N = 956).

Correlations of FSFI domain scores with sexual satisfaction scores for the SSc sample and the general population sample can be found in Table 5. For all sexual domains and for the FSFI total score, correlations with sexual satisfaction were substantially higher among women with SSc than among women from the population sample.

**Table 5:** Correlations of FSFI domain scores with sexual satisfaction scores among sexually active women with systemic sclerosis and sexually active women from a UK general population sample.

	CSRG Sample (N = 294)			UK Population Sample (N = 947)		
FSFI Domain	Correlation	95% CI	P value	Correlation	95% CI	P value
**Desire**	0.61	0.53–0.68	<0.001	0.42	0.37–0.47	<0.001
**Arousal**	0.68	0.61–0.74	<0.001	0.51	0.46–0.56	<0.001
**Lubrication**	0.50	0.41–0.58	<0.001	0.34	0.28–0.40	<0.001
**Orgasm**	0.70	0.64–0.75	<0.001	0.51	0.46–0.56	<0.001
**Pain**	0.42	0.32–0.51	<0.001	0.30	0.24–0.36	<0.001
**Total FSFI score**	0.74	0.68–0.79	<0.001	0.57	0.53–0.61	<0.001

## Discussion

This is the first study to compare rates of sexual activity and function in a sample of women living with a serious chronic disease to women from a general population sample. Although the SSc sample was from Canada and the general population sample was from the UK, the results were sufficiently robust to be confident that, controlling for age and marital status, women with SSc were significantly less likely to be sexually active and significantly more likely to be sexually impaired than women from a general population sample. This finding consistently held across groups stratified by age and marital status. When women with SSc and women from the general population with similar sexual impairment scores were compared, women with SSc had significantly worse lubrication and pain scores. Overall sexual satisfaction was more highly associated with impairment ratings among women with SSc compared to women from the general population sample. There are many factors that may influence sexual satisfaction, some of which are related to health status and impairment and others that are not. This finding suggests that factors related to sexual impairment were more important in the scope of overall sexual satisfaction in SSc than in the general population, whereas other factors may play a larger role.

Low rates of sexual activity and high rates of sexual impairment are commonly reported in women with chronic diseases [18], including SSc [11], [12], [19]–[23]. However, no previous studies had compared rates to those from a general population sample using a validated method of assessment. Thus, the extent to which rates among women with chronic diseases, including SSc, were different from the general population was not clear.

We know of two large general population studies that have used the FSFI to separately estimate rates of sexual activity and impairment [9], [10]. One of these two studies comprised the sample of women in the UK that was used in the present study [9]. In this sample, 64% of women were sexually active, and among sexually active women, 44% were sexually impaired, resulting in 36% who were active without impairment. The other large population study was a sample of 3,205 women from the Boston metropolitan area [10]. In this study, 51% were sexually active, and 38% of those sexually active were sexually impaired, resulting in 32% sexually active without impairment [10]. In both general population studies, rates of sexual activity and impairment were strongly associated with age and marital status [9], [10]. Neither study, however, published data in a form that allowed direct comparison of published results, disaggregated by age and marital status, between women from the general population and women with scleroderma. We were able to obtain the original data from the twins study for the present study because the data were publically available in a post-study repository.

An important contribution of the current study was that it is the first study to directly compare sexual activity and impairment among women with a chronic medical disease to women from a general population sample, using original data from both samples and controlling for both age and marital status. Another important contribution is that it directly compared sexual functioning domains among women with SSc to general population women. In SSc, several previous studies have suggested that rates of sexual impairment might be high using different instruments and methods, and have suggested factors that may be related [11], [12], [19]–[23]. No previous studies, however, used a validated measure to compare domains of sexual function that may be problematic for women with SSc. The finding of the present study that lubrication is a key problem driving impairment in SSc is consistent with literature suggesting that vaginal dryness is commonly reported among women with SSc, and is linked to sexual impairment [11], [13], [23]. In addition, the finding that pain was also an important factor driving impairment in SSc is consistent with previous research, which found that over 60% of sexually active female SSc patients report experiencing pain during sexual activity, and almost 40% report experiencing pain after sexual activity [12].

In addition to symptomatic treatments for SSc symptoms, including vasodilators for Raynaud's syndrome and finger ulcers, proton pump inhibitors and promotility agents for gastric reflux, and general analgesia (e.g., acetaminophen, anti-inflammatories when not contra-indicated, and narcotics if necessary), several authors have suggested steps that women with SSc can take that may reduce their pain and discomfort during sexual activity [11], [13], [14]. For instance, a water-based lubricant may be useful to reduce vaginal dryness and dyspareunia [11], [13], [14], [19], [20], [23]. A warm bath before sexual activities, attempting alternative sexual positions, and using pillows may reduce the effects of painful joints [11], [14], [20], [22]. Good communication during sexual activity has also been emphasized so that partners are aware of what is pleasurable and painful [14]. It is also possible that sexual function could be improved through range of motion exercises to reduce joint pain and stiffness prior to sexual activity, massage or exercises to lessen mouth tightening and improve mouth function, and massage or gentle manual stretching to lessen vaginal tightness. The degree to which these suggestions are effective in reducing barriers to sexual activity and enhancing the sexual experience of women with SSc, however, has not been tested.

There are a number of limitations that should be considered in interpreting the results of our study. First, it was cross-sectional and conducted with a convenience sample of patients enrolled in the CSRG Registry. Patients with very severe SSc who were too sick to participate, as well as those who may have died earlier in their disease course, are not enrolled in the Registry, which may result in an over-representation of healthier patients. Although approximately 80% of approached patients enroll in the Registry, data on patients who do not participate are not available. Second, the non-medical, population sample of the Adult Twins Registry is from a different country than that of the CSRG Registry, which could influence comparability. Third, the non-medical, population sample was from a twin registry. However, there is no reason to expect that a twin sample would bias results, and there are no other readily available population samples to make any attempt at benchmarking the levels of activity and impairment from SSc. Additionally, the Adult Twins Registry has been shown to be representative of the general population for a wide range of lifestyle and sexual behavioural factors [26], [27]. Thus, although this comparison does not permit strong claims about precise estimates of the risk of non-activity and dysfunction in SSc, it provides a general context and allows for a global understanding of the degree of sexual impairment from SSc compared to non-medically ill women. In both studies, women indicated if they had been sexual active or not, but no definition for the term “sexual activity” was provided. Thus, it is possible that this term may have been interpreted differently by different women. For instance, it is possible that some may have considered aspects such as hugging, snuggling, kissing and touching to be sexual activities, whereas others may have considered sexual activity to be defined solely as some form of penetration. Another limitation is that the 9-item version of the FSFI has not been specifically validated, although it has been used previously in SSc [12]. Furthermore, we do not know to what degree having sexual problems may have influenced whether or not women were married, however this would apply across both samples in this study. Finally, while there were missing data in both samples, the proportion of missing data was very low in both samples (9% in SSc and 6% in the UK sample), and the relatively small differences in the characteristics of women with complete data and those with missing data were consistent across samples.

In summary, sexual impairment is a problem for many women living with scleroderma. Adjusting for age and marital status, women with SSc were less than half as likely to be sexually active and, among sexually active women, almost twice as likely to be sexually impaired than women in the general population. Overall, only 16% of women with SSc were sexually active without impairment, compared to 36% in the general population. Controlling for total FSFI scores, women with SSc had significantly worse pain and lubrication scores than women in the general population. Research is needed to develop interventions to target pain and lubrication problems, specifically, and to improve overall sexual functioning among women with this disease.
